# BAY 60-6583 Enhances the Antitumor Function of Chimeric Antigen Receptor-Modified T Cells Independent of the Adenosine A2b Receptor

**DOI:** 10.3389/fphar.2021.619800

**Published:** 2021-03-12

**Authors:** Jiaxing Tang, Yan Zou, Long Li, Fengping Lu, Hongtao Xu, Pengxuan Ren, Fang Bai, Gabriele Niedermann, Xuekai Zhu

**Affiliations:** ^1^ Shanghai Institute for Advanced Immunochemical Studies, ShanghaiTech University, Shanghai, China; ^2^ School of Life Science and Technology, ShanghaiTech University, Shanghai, China; ^3^ Institute of Biochemistry and Cell Biology, Chinese Academy of Sciences, Shanghai, China; ^4^ University of Chinese Academy of Sciences, Beijing, China; ^5^ Department of Radiation Oncology, Faculty of Medicine, University of Freiburg, Freiburg, Germany; ^6^ German Cancer Consortium, Partner Site Freiburg and German Cancer Research Center, Heidelberg, Germany

**Keywords:** chimeric antigen receptor, BAY 60-6583, solid tumor, immunotherapy, immunosuppression

## Abstract

Chimeric antigen receptor (CAR) T cells are powerful in eradicating hematological malignancies, but their efficacy is limited in treating solid tumors. One of the barriers is the immunosuppressive response induced by immunomodulatory signaling pathways. Pharmacological targeting of these immunosuppressive pathways may be a simple way to improve the efficacy of CAR T cells. In this study, anti-CD133 and anti-HER2 CAR T cells were generated from healthy donors, and combination therapy using CAR T cells and small molecules targeting adenosine receptors was performed *in vitro* and *in vivo* with the goal of probing for potential synergistic antitumor activities. The adenosine A2b receptor agonist, BAY 60-6583, was found to significantly increase cytokine secretion of CD133-or HER2-specific CAR T cells when co-cultured with the respective target tumor cells. The *in vitro* cytotoxicity and proliferation of CAR T cells were also enhanced when supplied with BAY 60-6583. Furthermore, the combination with this small molecule facilitated the anti-HER2 CAR T cell-mediated elimination of tumor cells in a xenograft mouse model. However, the enhanced antitumor activities could not be suppressed by knockout of the adenosine A2b receptor in CAR T cells. Furthermore, mass spectrometry and computational methods were used to predict several potential alternative targets. Four potential targets (pyruvate kinase M (PKM), Talin-1, Plastin-2, and lamina-associated polypeptide 2) were captured by a photo-affinity probe, of which PKM and Talin-1 were predicted to interact with BAY 60-6583. Overall, our data suggest that BAY 60-6583 upregulates T cell functions through a mechanism independent of the adenosine A2b receptor.

## Introduction

Chimeric antigen receptors (CARs) are synthetic receptors that are transfected into T cells and direct them to target molecules on the tumor cell surface for the subsequent elimination of tumor cells (Newick et al., 2016). CARs consists of an extracellular single-chain variable fragment (scFv; which can specifically bind to a target on the tumor cell surface), a transmembrane spacer, and cytoplasmic signaling domains giving rise to T cell activation and proliferation (Newick et al., 2016; Sadelain, 2016). Unlike T cell receptors (TCRs), CARs recognize cell surface targets independent of antigen presentation by major histocompatibility complex (MHC) molecules. Therefore, CARs are not constrained by the patients’ human leukocyte antigens (HLAs) and can also identify tumors with low HLA expression. In addition, CAR T cells can recognize different types of target molecules, not only proteins but also carbohydrates and glycolipids typically expressed on the surface of tumor cells (Sadelain et al., 2009; Sadelain, 2016).

To date, CAR T cells have shown tremendous success in eradicating hematological malignancies (e.g., CD19-specific CARs in leukemia), but the clinical results in solid tumors are less encouraging (Newick et al., 2016; Martinez and Moon, 2019). There are at least three barriers that prevent successful eradication of solid tumors by CAR T cells: the identification of tumor-specific cell surface target molecules, T cell trafficking, and T cell dysfunction due to immunosuppression and excessive antigen exposure (Newick et al., 2016; Martinez and Moon, 2019). Recent research has suggested that targeting immunosuppressive mechanisms would indeed be a potential strategy to improve CAR T cell efficacy in the treatment of solid tumors.

There is evidence that adenosine can establish an immunosuppressive environment by downregulating the antitumor activity of effector T cells, recruiting immunosuppressive cells like CD4 + regulatory T cells (Tregs), and promoting angiogenesis to support tumor progression with the help of immunosuppressive cytokines produced by immunoregulatory cells. All of these adenosine-related immunosuppressive effects are driven by its activation of downstream adenosine receptors (Zou, 2005; Allard et al., 2016; Gilbert et al., 2019). The critical role of adenosine makes it a potential therapeutic target to substantially reduce immunosuppression in solid tumors.

There are four types of adenosine receptors: A1, A2a, A2b, and A3. The adenosine A2a receptor has been reported to mediate anti-inflammatory responses (Beavis et al., 2017; Kazemi et al., 2018; Sek et al., 2018), but the role of the other adenosine receptors in immunotherapy remains to be defined. Commercial compounds targeting various adenosine receptor subtypes as selective agonists and antagonists are readily available, and they are widely used to evaluate the role of these receptors in diseases (Kazemi et al., 2018; Sek et al., 2018). Therefore, these small molecules could be useful tools to analyze the function of adenosine receptors in CAR T cell therapy.

We screened a variety of small-molecule compounds that have been suggested to act either as agonists or as antagonists of one of the four receptors of the immunosuppressive adenosine molecule, using CD133-specific CAR T cells (Zhu et al., 2015; Zhu and Niedermann, 2015) and HER2-specific CAR T cells (Wang et al., 2020a; Wang et al., 2020b). The adenosine A2b receptor-selective agonist, BAY 60-6583, was selected for further studies because it significantly promoted CAR T cell cytokine secretion. BAY 60-6583 also enhanced the CAR T cell-mediated antitumor efficacy *in vitro* and *in vivo*. In addition, our data suggest that the enhancement of CAR T cells efficacy by BAY 60-6583 is independent of the adenosine A2b receptor, and we identified potential alternative functional targets in this study.

## Materials and Methods

### Reagents

The adenosine A1 receptor agonist *N* ^6^-cyclopentyladenosine (N6-CPA, purity > 98%) was obtained from Abcam (Shanghai, China) and dissolved in DMSO at 50 mM. The adenosine A1 receptor antagonist rolofylline (KW-3902, purity > 99%) was obtained from Tocris Bioscience (Bristol, United Kingdom) and dissolved in DMSO at 10 mM. The adenosine A2a receptor agonist CGS 21680 (purity > 98%) was obtained from Abcam and dissolved in DMSO at 10 mM. The adenosine A2a receptor antagonist SCH 58261 (purity > 99%) was obtained from Abcam and dissolved in DMSO at 10 mM. The adenosine A2b receptor agonist BAY 60-6583 (purity > 98%) was obtained from Tocris Bioscience and dissolved in DMSO at 20 mM. The adenosine A2b receptor antagonist PSB603 (purity > 98%) was obtained from Tocris Bioscience and dissolved in DMSO at 10 mM. The adenosine A3 receptor agonist piclidenoson (IB-MECA, purity > 97%) was obtained from Abcam and dissolved in DMSO at 10 mM. The adenosine A3 receptor antagonist MRE 3008F20 (purity > 98%) was obtained from Tocris Bioscience and dissolved in DMSO at 50 mM. The adenosine analog 5′-*N*-ethylcarboxamidoadenosine (NECA, purity > 99%) was obtained from Tocris Bioscience and dissolved in DMSO at 50 mM. All solutions were stored at –20°C and used within 1 year.

### Cells

The human glioma cell line U251 and the breast cancer cell lines MDA-MB-453 (HER2 positive, Supplementary Figure S1A) and MDA-MB-468 (HER2 negative, Supplementary Figure S1A) were purchased from the Cell Bank of the Chinese Academy of Sciences (Shanghai, China). The plasmids encoding human CD133 protein and piggyBac transposase were nucleofected into U251 cells to generate a CD133-overexpressing glioma cell line after puromycin selection (Supplementary Figure S1B). Then, the CD133-overexpressing U251 cells (U251-CD133OE) were nucleofected again with the plasmids expressing firefly luciferase and piggyBac transposase, followed by drug selection to obtain a luciferase-expressing tumor cell line (U251-CD133OE luc).

Similarly, other tumor cell lines were modified to obtain U251 WT luc, MDA-MB-453 luc, and MDA-MB-468 luc cells. G418 at 1 mg/ml was used to maintain luciferase expression.

### Generation and Expansion of Chimeric Antigen Receptor T Cells

The generation of CD133-specific CAR T cells and of HER2-specific CAR T cells has been described previously (Zhu et al., 2015; Zhu and Niedermann, 2015; Wang et al., 2020a; Wang et al., 2020b). In brief, AIM V™ (A3021002, ThermoFisher Scientific, Waltham, MA, United States) + 10% FBS (10099141C, ThermoFisher Scientific) were used to expand and culture CAR T cells.

Frozen human peripheral blood mononuclear cells (PBMCs) from healthy donors were purchased from AllCells (PB005F, Alameda, CA, United States). After thawing and resting for 1.5–2 h, 1.5 × 10^7^ PBMCs were resuspended in 100 μL of supplemented Nucleofector solution (Lonza; VPA-1002) and DNA mix (10 μg piggyBac transposon plasmid expressing the CAR and 5 μg piggyBac transposase plasmid) for one nucleofection reaction. Electroporation was performed with a Nucleofector™ II/2b device (Lonza) using the U-014 program. Electroporated cells were rested overnight before expansion. First expansion: Allogeneic PBMCs from four to five different donors were irradiated with 40 Gy and added to the T cell culture at a ratio of 10:1. In addition, the cell culture was supplemented with 50 ng/ml of anti-CD3 antibody (Miltenyi; 130-093-387) and 300 IU/ml of IL-2. Every 2–3 days, the medium was refreshed and fresh IL-2 was supplemented. Four days after stimulation, 0.5 μg/ml puromycin was added to select CAR-expressing T cells. Second expansion: On day 14, cells were restimulated as described above for the first expansion, and 0.5 μg/ml puromycin was added at the beginning to maintain CAR expression. CAR T cells were harvested on day 24, and CAR expression was measured (Supplementary Figure S1C) with an anti-c-Myc antibody (clone 9B11, Cell Signaling Technology, Boston, MA, United States).

### Generation and Expansion of Adenosine A2b Receptor Knockout Chimeric Antigen Receptor T Cells

Adenosine A2b receptor knockout CAR T cells were prepared using the same protocol as for the generation of conventional CAR T cells described above, but a different DNA mix (5 μg piggyBac transposon plasmid expressing the CAR, 5 μg piggyBac transposase plasmid, 5 μg Cas9-GFP plasmid, and 5 μg plasmid encoding sgRNA (2 + 6, 4 + 6, or 5 + 6) targeting the adenosine A2b receptor) was used for one nucleofection reaction (Hu et al., 2019).

sgRNA sequences:

sgRNA-2 GCT​GGT​CAT​CGC​CGC​GCT​TT
 sgRNA-4 GAC​ACA​GGA​CGC​GCT​GTA​CG
 sgRNA-5 GGA​CGC​GCT​GTA​CGT​GGC​GC
 sgRNA-6 GTG​CTG​GTG​TGC​GCC​GCG​GT

### Cytokine Secretion Assay

CAR T cells were cultured at 2 × 10^5^ cells/well in a 96-well plate in a volume of 200 μL per well. The small molecule or vehicle control was added at the same time. After 16 h, the supernatants of the cultures were collected, and cytokines (TNF-α, IFN-γ, and GM-CSF) were detected using AlphaLISA detection kits (TNF-α, AL208C; IFN-γ, AL217C; and GM-CSF, AL216C) from PerkinElmer, Waltham, MA, United States.

### Cytotoxicity Assay

Tumor cells stably expressing firefly luciferase were seeded in 96-well white microplates (PerkinElmer, Waltham, MA, United States). CAR T cells were added according to different effector-to-target cell (E:T) ratios. The small molecule or vehicle control was added at the same time. The cells were cultured in 200 μL of T cell medium without cytokines. After 24–48 h, 150 μg/ml D-luciferin K+ salt (PerkinElmer, 122799) was added, and the signals were read immediately using an EnSpire Multimode plate reader (PerkinElmer).

The percentage of target cell death was calculated according to the following formula:

Lysis(%)=(1−signal value of co - culture well/signal value of tumor cell alone well)×100.

### T Cell Proliferation Assay

Target tumor cells were irradiated with 70 Gy using an X-ray irradiator and then seeded overnight. On the next day, CAR T cells were labeled with 0.5 μM CFSE (carboxyfluorescein diacetate succinimidyl ester; 65-0850-84, ThermoFisher Scientific) following the manufacturer’s protocol. The labeled CAR T cells were co-cultured with target cells at the indicated E:T ratios. Then, BAY 60-6583 (final concentration 10 μM) or vehicle control was added to each group. Cells were cultured in T cell medium supplemented with 300 IU/ml of IL-2; the T cell concentration was 1 × 10^6^/ml. At the indicated time points, T cell proliferation was detected by flow cytometry, and the results were analyzed using FlowJo V-10 (BD Biosciences, Bedford, MA, United States).

### Treatment of Tumor-Bearing Mice

The animal experiments were approved by the Shanghai Administrative Committee for Laboratory Animals. Female NOD-PrkdcscidIl2rgnull/Shjh (NPSG) mice (four to eight weeks old) were injected subcutaneously into the right front flank with 3 × 10^6^ MDA-MB-453-luc cells suspended in phenol red-free Matrigel (Corning Incorporated, Corning, NY, United States). When the tumors reached a size of 100–150 mm^3^ (1 week after inoculation), 10 × 10^6^ anti-HER2 CAR T cells were intravenously injected into the tumor-bearing mice. After that, mice were intravenously treated with 20 μg BAY 60-6583 or vehicle control daily (3 mice per group). Tumor growth was measured directly using a digital caliper, and the tumor volume was calculated using the formula length × width^2^ × 0.5.

### Western Blot

Cells were thawed and washed twice with PBS, and then the cells were lyzed in RIPA buffer. SDS-PAGE was used to separate the proteins in the samples. The electrophoresed proteins were transferred onto a PVDF membrane using the semi-dry method (15 V, 35 min). After blocking with 5% milk for 1 h, the membranes were incubated with anti-adenosine A2b receptor (AAR-003, alomone labs) or anti-GAPDH (sc-32233, Santa Cruz Biotechnology) antibodies at 4°C overnight. Finally, the membranes were incubated with peroxidase-conjugated AffiniPure donkey anti-rabbit IgG (H + L) (JAC-711-035-152, Jackson, MS, United States) or peroxidase-conjugated AffiniPure donkey anti-mouse IgG (H + L) (JAC-715–035–150, Jackson, MS, United States) at room temperature for 1 h, and the blots were visualized with a Bio-Rad Imager (Bio-Rad, Hercules, CA, United States).

### Photo-Crosslinking Sample Preparation

T cells (5 × 10^7^) were seeded at 2 × 10^6^ cells/ml and activated by T Cell TransAct™ human (Miltenyi Biotec GmbH, Cologne, Germany) according to the manufacturer’s protocol. Cells were cultured in the dark and the photo-affinity probe was added at 10 μM. After overnight treatment, the cells were resuspended with pre-cooled PBS and irradiated for 5 min (*λ* = 350 nm). Then, the cells were lyzed in SDS buffer and sonicated 10 times (10 s on, 10 s off). The protein concentration was measured and adjusted to 0.7 mg/ml. Then, the protein sample was analyzed by liquid chromatography-mass spectrometry (LC-MS).

### Statistics

All the calculations were performed using GraphPad Prism V6.01. Statistical tests were performed as indicated in the figure legends with a *p*-value less than 0.05 considered as significant.

## Results

### BAY 60-6583 Significantly Increases the Cytokine Production of Chimeric Antigen Receptor T Cells

**FIGURE 1:**
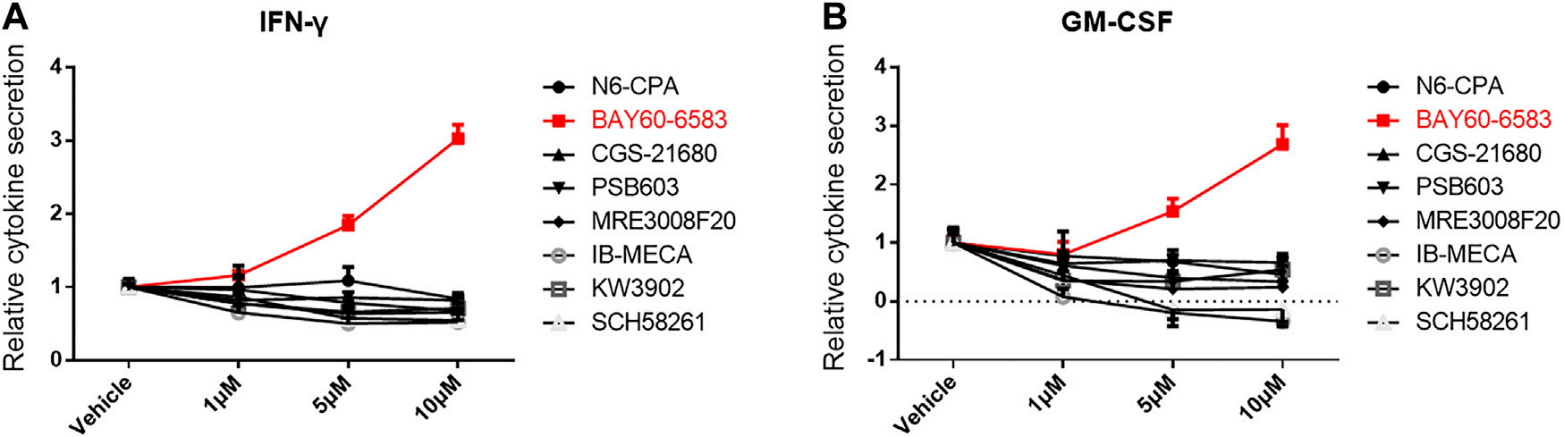
BAY 60-6583 enhances cytokine secretion of activated anti-CD133 CAR T cells. Anti-CD133 CAR T cells were co-cultured with U251-CD133OE cells, the effector-to-target cell (E:T) ratio was 4:1. Vehicle and eight small molecules targeting adenosine receptors were added at different final concentrations. After 16 h, the medium was collected and secretion of INF-γ **(A)** and GM-CSF **(B)** was detected using AlphaLISA kits. Data were normalized to vehicle control and are represented as the mean ± SD of triplicates from a representative experiment of *n* = 3 experiments.

To investigate the role of small molecules targeting adenosine receptors for regulating CAR T cell function, the adenosine A1 receptor agonist N6-CPA, the adenosine A1 receptor antagonist rolofylline (KW-3902), the adenosine A2a receptor agonist CGS 21680, the adenosine A2a receptor antagonist SCH 58261, the adenosine A2b receptor agonist BAY 60-6583, the adenosine A2b receptor antagonist PSB603, the adenosine A3 receptor agonist piclidenoson (IB-MECA), and the adenosine A3 receptor antagonist MRE 3008F20 were separately added to co-cultures containing anti-CD133 CAR T and U251-CD133OE cells. IFN-γ and GM-CSF secreted into the supernatant of the co-cultures were detected 16 h later. As shown in Figure 1, IFN-γ and GM-CSF secreted by anti-CD133 CAR T cells were significantly upregulated under BAY 60-6583 treatment. This result was surprising since BAY 60-6583 has been proposed to act as an adenosine A2b receptor agonist (Allard et al., 2016; Gao and Jacobson, 2019).

### BAY 60-6583-mediated Chimeric Antigen Receptor T cell enhancement requires T cell activation but is not dependent on Chimeric Antigen Receptor signaling

**FIGURE 2:**
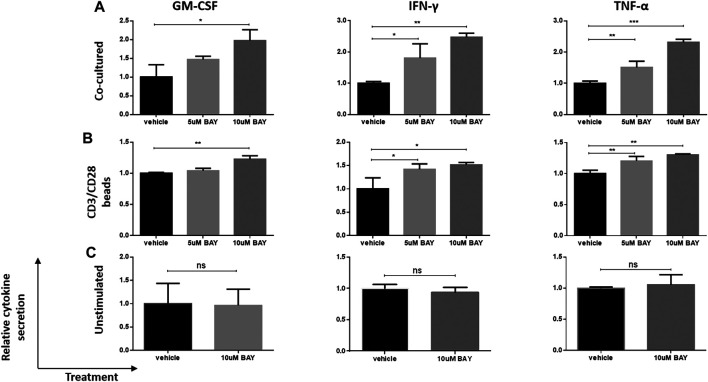
BAY 60-6583-mediated CAR T cell enhancement requires T cell activation. Anti-CD133 CAR T cells (1 × 10^6^) were activated by co-incubation with 2.5 × 10^5^ U251-CD133OE cells **(A)** or CD3/CD28 beads **(B)**; unstimulated CAR T cells **(C)** were used as control. Vehicle and BAY 60-6583 were added at different final concentrations. After 16 h, the medium was collected and the secretion of GM-CSF, INF-γ, and TNF-α was detected using AlphaLISA kits. Data were normalized to vehicle control and are represented as the mean ± SD of triplicates from a representative experiment of *n* = 4 experiments. *p < 0.05, **p < 0.01, ***p < 0.001 by 1-way ANOVA; ns, not significant.

Then, we investigated whether the enhancement of cytokine secretion was CAR dependent. Unstimulated, CD3/CD28 beads- (TCR signal) or U251-CD133OE tumor cell-activated (CAR signal) anti-CD133 CAR T cells were treated with BAY 60-6583 or vehicle control for 16 h. Then, the medium was collected for cytokine assays. Compared with the vehicle control, T cells activated by the CAR signal (Figure 2A) or TCR signal (Figure 2B) secreted more GM-CSF, IFN-γ, and TNF-α in the presence of BAY 60-6583; however, no significant differences were found in unstimulated samples (Figure 2C). These results show that the upregulation of cytokine production mediated by BAY 60-6583 requires T cell activation and that CAR signaling is not necessary in this process.

### BAY 60-6583 specifically improves the in vitro antitumor activity of both anti-CD133 Chimeric Antigen Receptor and anti-HER2 Chimeric Antigen Receptor T cells

**FIGURE 3:**
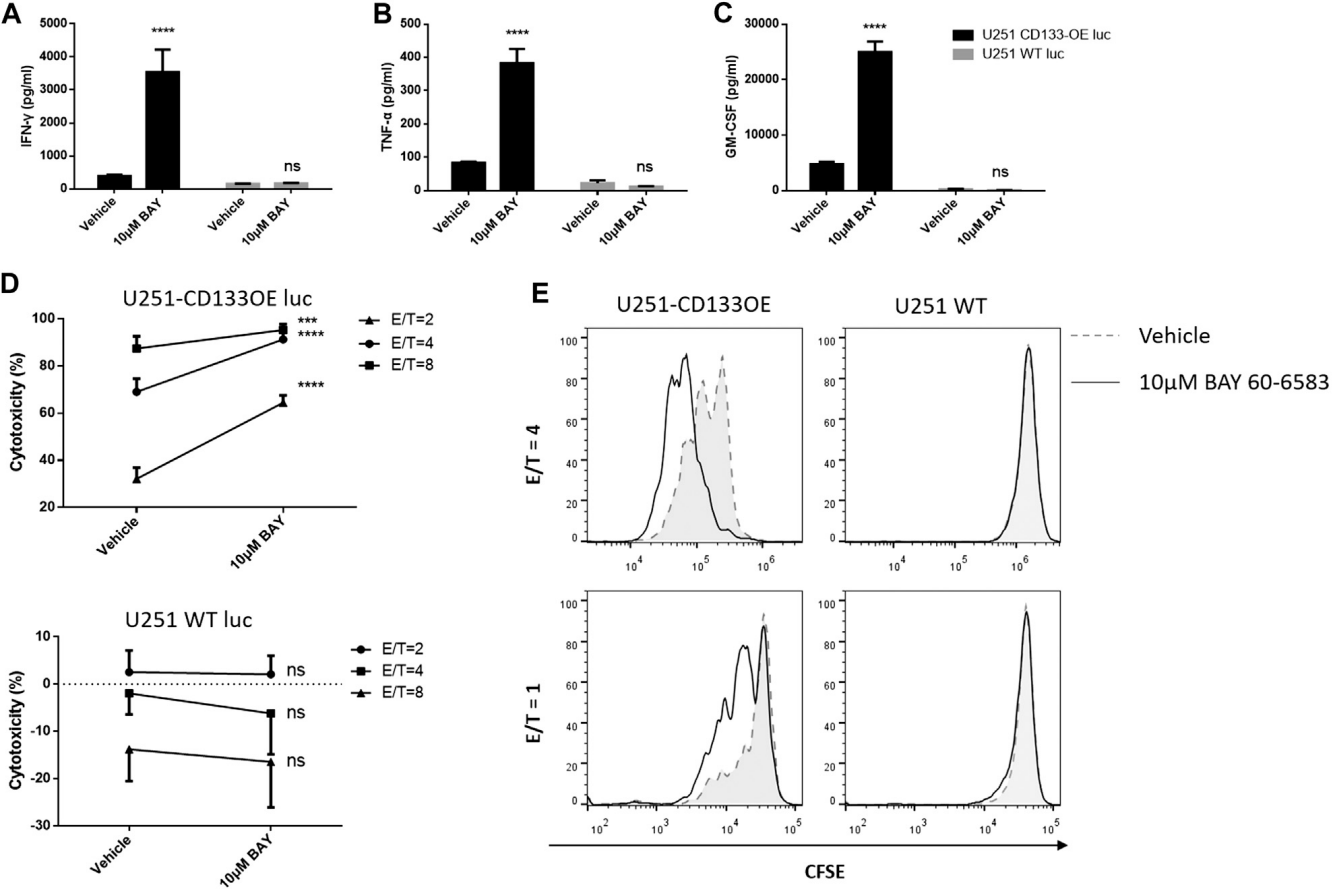
BAY 60-6583 specifically improved activities of CD133-CAR T cells. **(A–D)** Anti-CD133 CAR T cells were co-cultured with U251-CD133OE luc cells or U251 WT luc cells at different E:T ratios. Co-cultures were performed in the presence of vehicle or BAY 60-6583. After 16 h, the medium was collected, and the secretion of INF-γ **(A)**, TNF-α **(B)**, and GM-CSF **(C)** was detected using AlphaLISA kits (results for the E:T ratio of 4:1 are shown). **(D)** At 48 h after co-culture, cytotoxicity was determined by detecting the bioluminescence signal. Data are represented as the mean ± SD of triplicates from a representative experiment of *n* = 5 experiments. ****p < 0.0001 by 2-way ANOVA; ns, not significant **(E)** CFSE-labeled CAR T cells were co-cultured with irradiated U251-CD133OE cells in the presence of BAY 60-6583 or vehicle control. After 120 h, flow cytometry was used to analyze cell proliferation. Results shown are from a representative experiment of *n* = 4 experiments.

Next, we explored whether BAY 60-6583 enhances the antitumor activity of CAR T cells *in vitro* and, if so, whether this is CAR dependent. Anti-CD133 CAR T cells were incubated with target tumor U251-CD133OE cells; wild-type U251 cells were used as non-target controls. Similarly, anti-HER2 CAR T cells were activated by HER2-positive MDA-MB-453 cells, while HER2-negative MDA-MB-468 cells served as non-target controls. BAY 60-6583 and vehicle control were added at the beginning of the co-culture. Cytokine production by the CAR T cells measured using the AlphaLISA kit and cytotoxic destruction of the tumor cells determined by bioluminescence were assessed at the time points indicated in the legend to Figure 3. In the presence of BAY 60-6583, a higher level of cytokine production was observed for anti-CD133 (Figures 3A–C) and anti-HER2 (Supplementary Figure S2A–C) CAR T cells following antigen-specific stimulation, while no changes were observed when the CAR T cells were incubated with non-target tumor cells. Consistently, the tumor cell-killing capacity of CAR T cells was also enhanced when BAY 60-6583 was added and the viability of non-target tumors was not affected (Figure 3D and Supplementary Figure S2D).

In addition, BAY 60-6583 treatment also enhanced the proliferative ability of CAR T cells when stimulated by target tumor cells. CD133-specific and HER2-specific CAR T cells were labeled with CFSE and incubated with irradiated tumor cells. IL-2 was used to keep the T cells alive. When activated by target tumor cells, the CFSE profiles indicated faster cell division under compound treatment compared to vehicle control, while the CFSE peak remained unshifted when the CAR T cells were co-cultured with non-target cells, indicating that the CAR T cells did not divide without contact to target-expressing tumor cells with or without BAY 60-6583 (Figure 3E, Supplementary Figure S2E). Taken together, these results revealed that BAY 60-6583 improved the antitumor effects of CAR T cells in contact with target-expressing tumor cells *in vitro*, including upregulating cytokine production, improving tumor killing, and promoting CAR T cell proliferation.

### BAY 60-6583 reversed the impaired function of Chimeric Antigen Receptor T cells in an immunosuppressive environment *in vitro*

**FIGURE 4:**
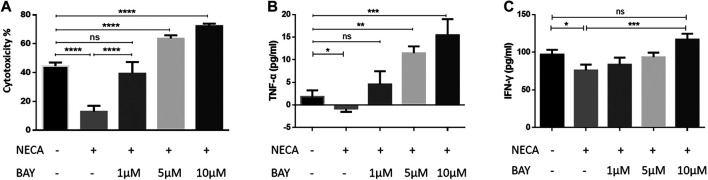
BAY 60-6583 enhanced CD133 CAR T cell function in a suppressive environment caused by an adenosine analog. Of the adenosine analog NECA, 1 μM was used to mimic an adenosine-enriched tumor microenvironment in the anti-CD133 CAR T cell co-culture system (E:T ratio of 2:1). After 4 h of NECA treatment, BAY 60-6583 was added. Bioluminescence was detected 48 h later for cytotoxicity analysis **(A)**, and the supernatant was collected for cytokine secretion detection **(B, C)**. *p < 0.05, **p < 0.01, ***p < 0.001, ****p < 0.0001 by 1-way ANOVA; ns, not significant. Data are represented as the mean ± SD from a representative experiment of *n* = 5 experiments.

Adenosine is an immunosuppressive molecule and works by activating adenosine receptors (Zou, 2005; Allard et al., 2016; Gilbert et al., 2019). As BAY 60-6583 has been considered as a selective agonist of the adenosine A2b receptor, we were wondering whether it would nevertheless enhance CAR T cell functions in an adenosine-rich immunosuppressive environment. To mimic an adenosine-rich microenvironment, 1 μM of the adenosine analog NECA was added to the anti-CD133 CAR T co-culture system 4 h before BAY 60-6583 was added (Beavis et al., 2017). Suppressed CAR T cell functions were observed in co-cultures with NECA alone, which included limited tumor killing (Figure 4A) and decreased TNF-α and IFN-γ production by the CAR T cells (Figures 4B,C). However, NECA-mediated suppressed tumor killing and TNF-α secretion were restored under 1 μM BAY 60-6583 treatment, and anti-CD133 CAR T cells even showed enhanced activity in the presence of higher concentrations of BAY 60-6583 (Figures 4A,B). BAY 60-6583 at 10 μM also restored the reduced IFN-γ secretion (Figure 4C). Taken together, these data revealed that BAY 60-6583 worked well in an adenosine-rich immunosuppressive environment and that it can reverse the suppressed antitumor activity of CAR T cells mediated by adenosine. This suggests the hypothesis that BAY 60-6583 might enhance CAR T cell functions independent of the adenosine A2b receptor.

### BAY 60-6583 administration enhanced the *in vivo* function of anti-HER2 Chimeric Antigen Receptor T cells

**FIGURE 5:**
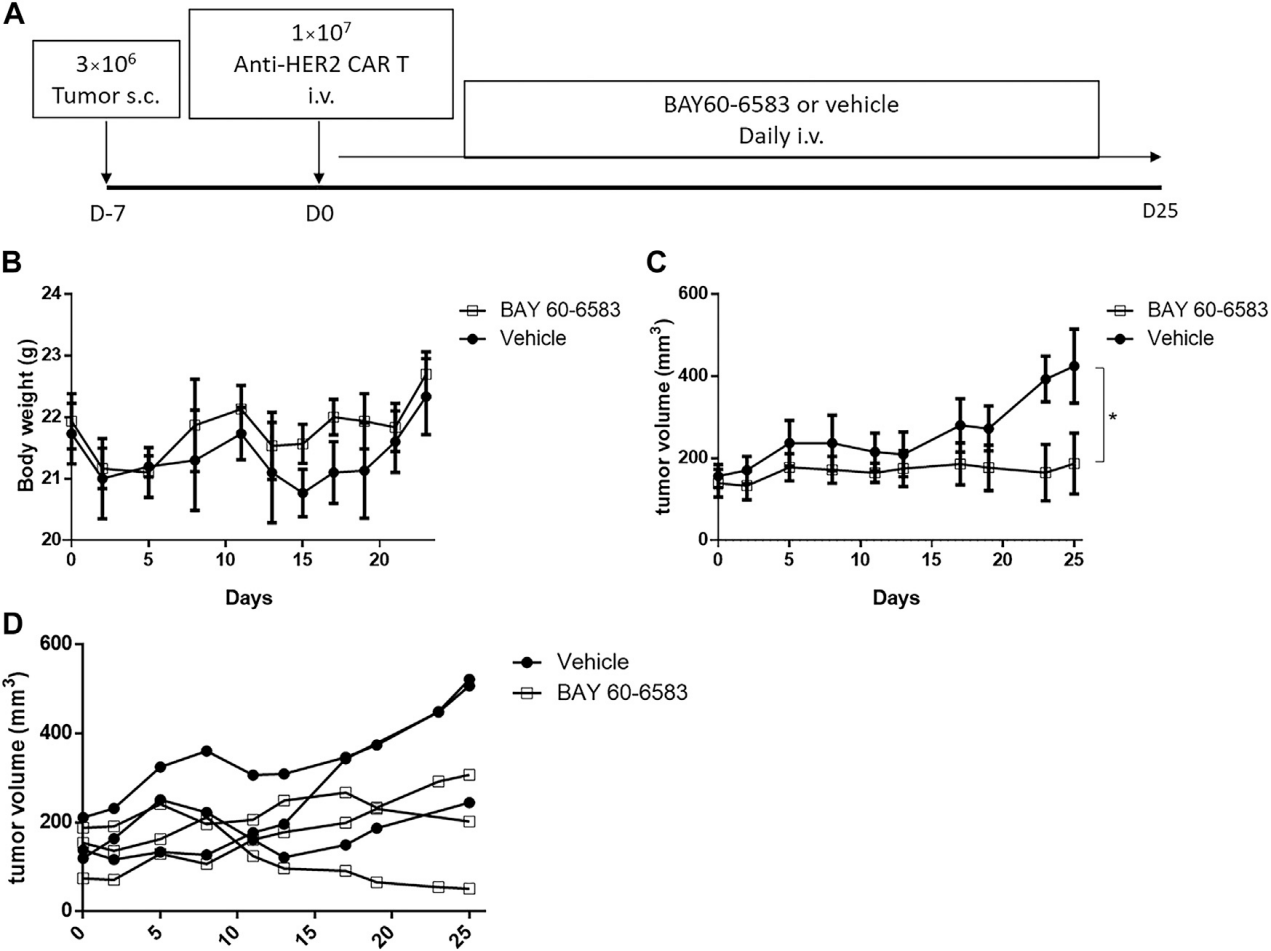
BAY 60-6583 improved the *in vivo* function of anti-HER2 CAR T cells **(A)** Overview of the experimental workflow **(B)** Body weight of vehicle-treated and BAY 60-6583-treated mice (*n* = 3 in each group) **(C)** Tumor volumes in anti-HER2 CAR T cell-treated mice receiving 20 μg BAY 60-6583 or vehicle control (*n* = 3 in each group). **(D)** Individual tumor growth curves are shown for each mouse presented in **(C)**. **(B)** Data are represented as the mean ± SD. **(C)** Data are presented as the mean ± SEM. *p < 0.05 by 2-way ANOVA.

Then, we investigated the functionality of CAR T cells *in vivo* in the presence of BAY 60-6583. NPSG mice were injected with luciferase-expressing MDA-MB-453 cells subcutaneously on day –7, and anti-HER2 CAR T cells were injected intravenously on day 0. After CAR T cell treatment, 20 μg BAY 60-6583 was administered intravenously daily (Figure 5A). BAY 60-6583 injection did not cause any apparent toxicity (Figure 5B). Compared to the vehicle control, daily treatment with 20 μg BAY 60-6583 had a stronger tumor-suppressive effect (Figures 5C,D). Taken together, the data indicate that BAY 60-6583 can enhance the antitumor activity of CAR T cells also in tumor models *in vivo*.

### The Chimeric Antigen Receptor T cell-enhancing effect of BAY 60-6583 is independent of the adenosine A2b receptor

As mentioned above, the adenosine A2b receptor has been proven to be an immunosuppressive target in many studies, and the receptor is expressed on effector T cells (Kazemi et al., 2018; Sek et al., 2018). Therefore, the presence of its selective agonist BAY 60-6583 in tumor/CAR T cell co-cultures should not show an enhanced immune response. We were therefore wondering whether these interesting effects were caused by A2b receptor-independent mechanisms.

**FIGURE 6:**
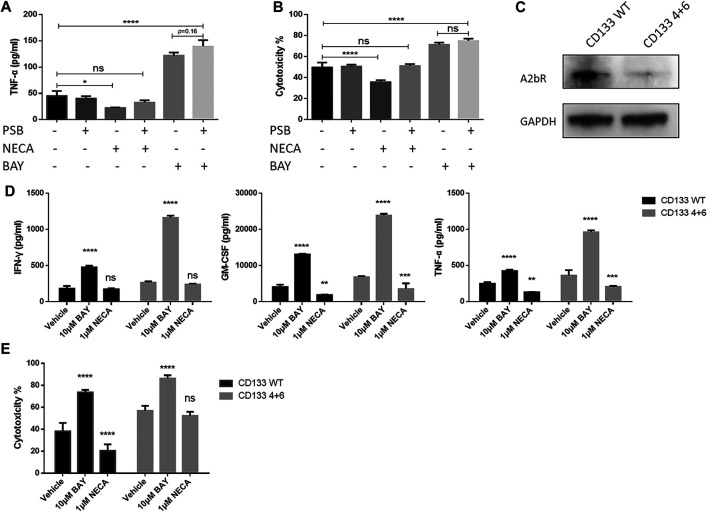
CAR T cell enhancement by BAY 60-6583 in CD133 CAR T cells seems not to be dependent on the adenosine A2b receptor. **(A, B)** Blocking of the adenosine A2b receptor cannot inhibit the enhanced antitumor activities induced by BAY 60-6583. In the anti-CD133 CAR T co-culture system (E:T ratio of 4:1), the adenosine A2b receptor was blocked by 1 μM PSB603 1 h before 10 μM BAY 60-6583 or 1 μM NECA was added. 24 h later, TNF-α production **(A)** and tumor lysis **(B)** were analyzed **(C)** The expression of the adenosine A2b receptor on conventional (WT) and A2b receptor knockout (4 + 6) anti-CD133 CAR T cells was determined by Western blot. Data from a representative experiment of *n* = 4 experiments **(D, E)** BAY 60-6583-induced enhancement of CAR T cell-mediated antitumor activities were not influenced by adenosine A2b receptor deficiency. Conventional and A2b receptor knockout anti-CD133 CAR T cells were co-cultured with U251-CD133OE luc cells at an E:T ratio of 4:1. After treatment with vehicle control or BAY 60-6583 for 24 h, cytokine secretion in the supernatant was detected **(D)**; 48 h later, cytotoxicity was assessed **(E)**. *p < 0.05, **p < 0.01, ***p < 0.001, ****p < 0.0001 by 1-way **(A, B)** or 2-way ANOVA **(D, E)**. Data are represented as the mean ± SD from a representative experiment of *n* = 3 experiments **(A, B, D, E)**.

At first, we designed an experiment using a selective antagonist targeting the adenosine A2b receptor to block the receptor and analyzed the effects of BAY 60-6583. The adenosine A2b receptor on the anti-CD133 CAR T cells was blocked by 1 μM PSB603 (Wei et al., 2013; Kaji et al., 2014) (an adenosine A2b receptor-selective antagonist) 1 h before BAY 60-6583 or NECA treatment in the anti-CD133 CAR T co-culture system. Then, 24 h later, TNF-α production and tumor cell lysis were analyzed. Blocking the adenosine A2b receptor by the selective antagonist did not inhibit the enhanced therapeutic response mediated by BAY 60-6583, but the immunosuppressive effects caused by NECA were restored (Figures 6A,B). These data indicated that the adenosine A2b receptor acted as an immunosuppressive target in an adenosine-enriched tumor microenvironment, because blocking this receptor with an antagonist repaired the suppressive effects caused by NECA. And most importantly, the data suggested that the adenosine A2b receptor might play a very limited role in the enhancement of the CAR T cell effects induced by BAY 60-6583.

To confirm the conclusions from the adenosine A2b receptor blocking model, we generated adenosine A2b receptor knockout (KO) anti-CD133 (Figure 6C and Supplementary Figure S1C) and anti-HER2 (Supplementary Figure S1C, S3A) CAR T cells using dual sgRNA-directed CRISPR/Cas9, and analyzed the antitumor activities of WT and KO (named after the sgRNAs used in production) CAR T cells in the presence or absence of BAY 60-6583. Under BAY 60-6583 treatment, more cytokine secretion (Figure 6D and Supplementary Figure S3C) and enhanced tumor killing ability (Figure 6E and Supplementary Figure S3B) were still observed in these receptor-deficient CAR T cells. Therefore, we concluded that BAY 60-6583 seems to have multiple targets and that its regulatory roles in CAR T cells when targeting tumor cells were achieved independent of the adenosine A2b receptor.

### Potential Targets Identified With a Photo-Affinity Probe

**FIGURE 7:**
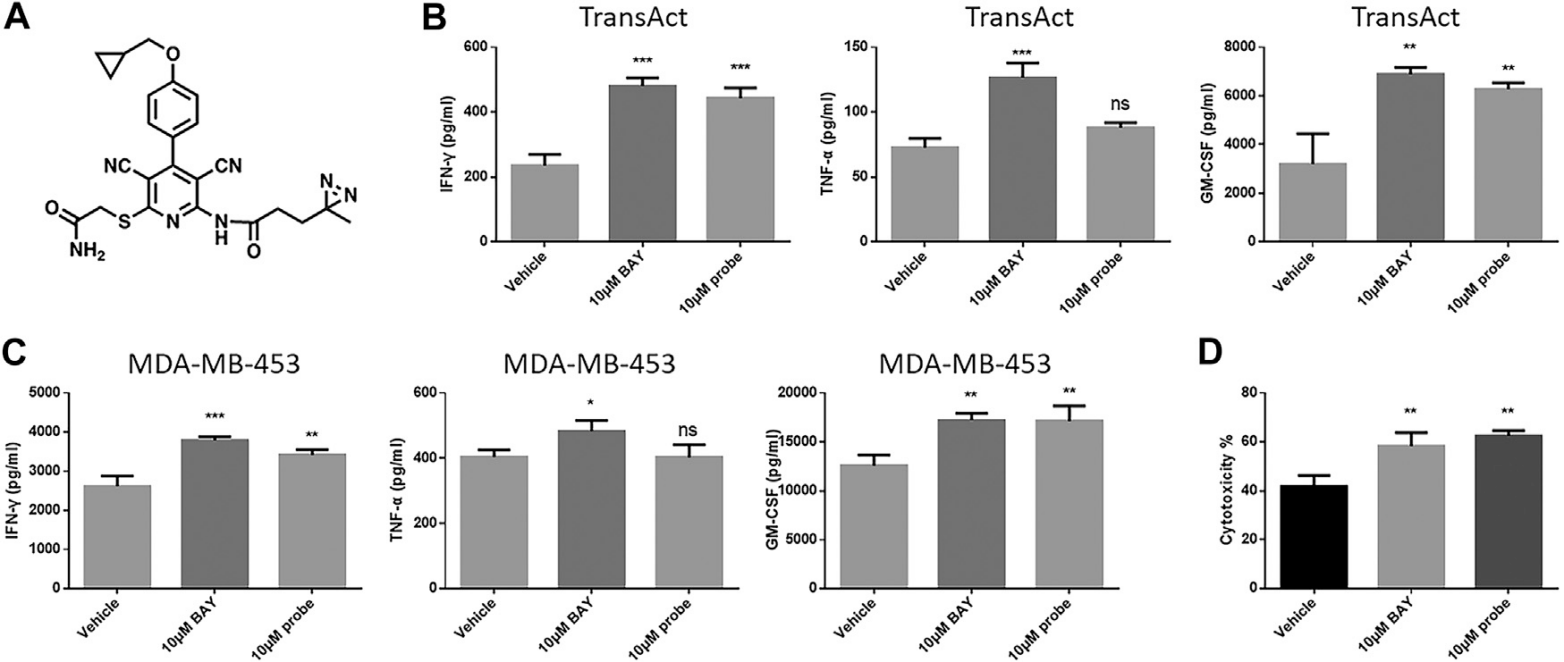
The photo-affinity probe enhanced CAR T cell effects similarly to unmodified BAY 60-6583 **(A)** The structure of the photo-affinity probe **(B–D)** Anti-HER2 CAR T cells (1 × 10^5^) were activated by TransAct **(B)** or 4 × 10^5^ MDA-MB-453 luc cells **(C, D)**. Probe, BAY 60-6583, or vehicle control was added at the beginning. After 16 h, the medium was collected, and the secretion of INF-γ, TNF-α, and GM-CSF was detected using AlphaLISA kits **(B, C)**; 48 h later, cytotoxicity was assessed **(D)**. Data are represented as the mean ± SD from a representative experiment of *n* = 3 experiments. *p < 0.05, **p < 0.01, ***p < 0.001 by 1-way ANOVA; ns, not significant.

To explore alternative candidate targets of BAY 60-6583, a probe with a photo-affinity crosslinker was designed and synthesized (Figure 7A). We first tested the effect of this probe on CAR T cell functionality. Anti-HER2 CAR T cells were activated by MDA-MB-453 tumor cells or TransAct, which provides primary and co-stimulatory signals for T cell activation, and cytokine secretion and cytotoxicity were assessed under compound treatment. Although the photo-affinity probe showed no effect on TNF-α secretion, it upregulated the production of IFN-γ and GM-CSF (Figures 7B,C) and facilitated anti-HER2 CAR T cell-mediated tumor cell killing (Figure 7D). Thus, the photo-affinity probe enhanced CAR T cell functions like the BAY 60-6583 compound.

Since the CAR T cell-enhancing effect of the photo-affinity probe was proven to be similar to that of BAY 60-6583, it was then incubated with TransAct-stimulated T cells to bind to potential targets. After overnight incubation, the photo-activated moiety was covalently crosslinked with potential target proteins by UV irradiation. Next, the cells were lyzed and labeled proteins were analyzed by LC-MS. Linked proteins were identified by increased molecular weight and are listed in Supplementary Table S1.

Most of the proteins listed are cytoskeleton-related proteins and are essential for the basic activities of cells. These proteins are highly enriched in cells, but there is no evidence that they are involved in the specific regulation of immune cell function (Supplementary Table S1). Besides this, we still found some proteins of interest with potential T cell-regulatory activity. This experiment revealed some candidates, but more evidence was needed to determine the likely target(s) of BAY 60-6583.

### Evaluating and Searching for Potential Targets Through Calculation Methods

**FIGURE 8:**
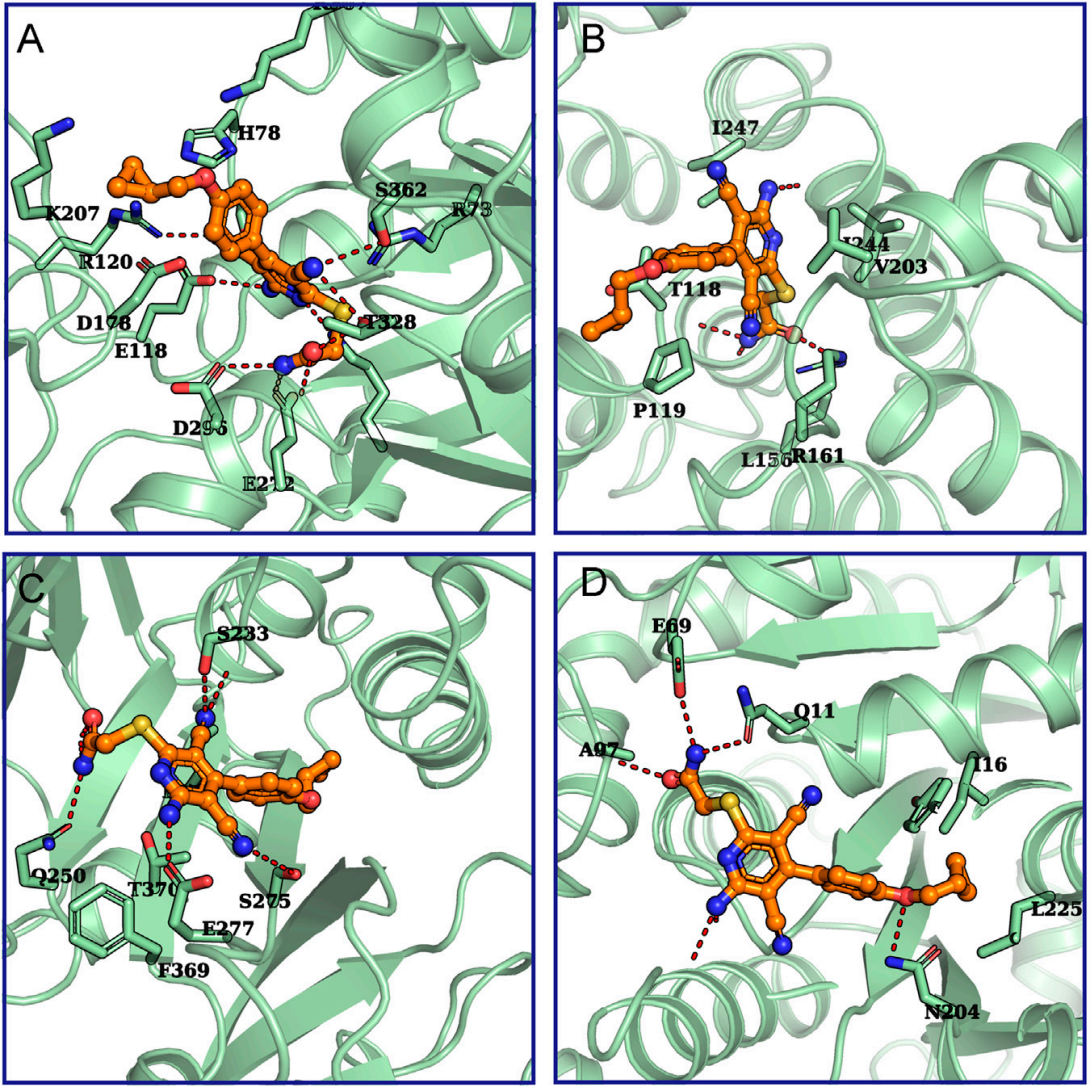
The predicted binding modes for BAY 60-6583 and potential targets. The interactions between **(A)** Annexin A5 (UniProtKB ID: P08758), **(B)** PKM (UniProtKB ID: P14618), **(C)** Talin-1 (UniProtKB ID: Q9Y490), or **(D)** tubulin beta-2A (UniProtKB ID: Q13796) with BAY 60-6583 were predicted. Green cartoons represent proteins. The orange stick-ball model is BAY 60-6583. The red dash lines are hydrogen bonds formed between the ligand and proteins.

In order to further evaluate the possibility that the proteins found by LC-MS were potential targets of BAY 60-6583, computational methods were used to predict and assess the binding between BAY 60-6583 and these proteins. Firstly, we used ITASSER (Yang et al., 2015), a well-known protein structure modeling method, to build protein structures for the proteins whose experimental structures were unavailable, followed by further structural refinement by using the Protein Preparation Wizard in Maestro-v12.1 (Sastry et al., 2013). All the related parameters were set as default. Of note, five of the 18 candidate proteins failed to be modeled due to the low homologies with templates or bad qualities of the generated structures. Then, molecular docking simulations were performed to assess the binding ability of BAY 60-6583 to these protein structures by using Glide-v8.4 (Friesner et al., 2004). To fully investigate the surface or potential binding sites of these proteins, multiple active sites being evenly distributed around the protein structures were defined for each protein. Next, docking simulations were run site by site for each protein. Finally, the site where the lowest energy binding mode of BAY 60-6583 for interaction was selected to assess the binding ability of the compound to the protein. All the results were collected in Supplementary Table S1 and the predicted binding modes for small-molecule and possible targets are shown in Figure 8. By considering both the predicted interaction modes of BAY 60-6583 to the candidate proteins and the gene functions related to T cells, PKM and Talin-1 were finally selected as the possible interacting targets of BAY 60-6583.

## Discussion

In this study, we screened several agonists and antagonists of the different known receptors of the immunosuppressive molecule adenosine. Surprisingly, the only small molecule that turned out to enhance the functionality of CAR T cells was BAY 60-6583, which has been suggested to act as an agonist of the adenosine A2b receptor. This counterintuitive finding can be explained by our additional data indicating that the CAR T cell-enhancing effects of BAY 60-6583 are independent of the adenosine A2b receptor. This is the first study showing that BAY 60-6583 can enhance the functionality of CAR T cells.

Pharmacological targeting of immune checkpoints combined with CAR T cells is a promising strategy for tumor treatment. Adenosine receptors are considered as important “immune checkpoints” because they are involved in tumor progression and functional T cell exhaustion (Zou, 2005; Allard et al., 2016; Gilbert et al., 2019), which has led to great interest in testing the function of molecules that target adenosine receptors in CAR T cells.

BAY 60-6583 is an agonist that targets the immunosuppressive adenosine A2b receptor, and it has been reported that activating the adenosine A2b receptor results in anti-inflammatory effects, like creating a tumor-promoting microenvironment, supporting tumor metastasis, and inducing immunosuppressive cells (Allard et al., 2016; Gao and Jacobson, 2019). However, we observed the opposite (immunostimulatory instead of suppressive) effect in this study, which might be the result of BAY 60-6583 binding to alternative targets rather than the adenosine A2b receptor, as reported in previous work (Schiedel et al., 2013; Borg et al., 2017). Neither blocking the adenosine A2b receptor by a selective antagonist nor knocking out the receptor in our CAR T cells could inhibit the enhanced antitumor activity mediated by BAY 60-6583. Therefore, we believe that the crucial role of BAY 60-6583 in CAR T cells is achieved through an adenosine A2b receptor-independent mechanism.

The exact functional targets are not clear, but we identified some potential candidates. The photo-affinity probe with biological activity was synthesized and incubated with activated CAR T cells to capture potential targets. Eighteen proteins were captured and identified through LC-MS, and then computational methods were used to evaluate the binding ability of our compound to these potential targets. Among these 18 different captured proteins, four candidate proteins (PKM, Talin-1, Plastin-2, and lamina-associated polypeptide 2) are reported to be involved in the development and function of immune cells. From molecular docking simulations, two of them (PKM and Talin-1) emerged to be the possible interacting targets of BAY 60-6583.

Pyruvate kinase is a glycolytic enzyme that catalyzes the transfer of phosphoenolpyruvate to pyruvate (Angiari et al., 2020), and pyruvate kinase isoform M2 (PKM2) is reported to be related to immune responses. PKM2 is suggested to play an important role in the differentiation and development of Th1 and Th17 cells, and targeting it can inhibit the pathogenicity of CD4^+^ cells (Kono et al., 2019; Angiari et al., 2020). In addition, PKM2 is reported to induce the expression of the immune checkpoint protein PD-L1 (Palsson-McDermott et al., 2017; Deng et al., 2018).

Talin-1 is a cytoskeletal protein that plays a role in lymphocyte cell-cell contacts (Bluggel et al., 2011). Talin-1 connects integrin to the actin cytoskeleton and is involved in cell adhesion (Manevich et al., 2007; Bluggel et al., 2011). A study has revealed that Talin-1 can influence Th cell differentiation (Meli et al., 2016).

Plastin-2 is reported to be related to T cell activation and functional activity. Plastin-2 is an actin-bundling cytoplasmic protein that is phosphorylated when T cells are stimulated via CD3 in combination with CD28 or CD2 (Wabnitz et al., 2007; Wabnitz et al., 2017). It participates in the process of T cell activation and transports activation molecules to the cell surface (Wabnitz et al., 2007). It helps form stable T cell/antigen-presenting cell contacts and is involved in synaptic maturation and stability (Wabnitz et al., 2007; Wabnitz et al., 2017). Reduced cytokine production, limited proliferation ability, and inhibited target cell killing are observed in Plastin-2 deficient T cells (Morley, 2013; Wabnitz et al., 2017).

Lamina-associated polypeptide 2 (isoforms beta/gamma) can be cleaved into thymopoietin, which is a thymic hormone that is reported to induce early T lymphocyte differentiation and affect T cell functions (Goldstein and Audhya, 1985; Lunin and Novoselova, 2010). Thymopentin (TP5), a pentapeptide corresponding to residues 32–36 of thymopoietin, has been characterized as an immunomodulator that can be used to treat immune deficiencies and imbalances. All these molecules play an immune-balancing role in inflammatory disease (Goldstein and Audhya, 1985; Gonser et al., 1999).

In this study, CAR T cells combined with the adenosine A2b receptor agonist BAY 60-6583 showed enhanced antitumor activities compared to CAR T cells alone. However, the adenosine A2b receptor apparently does not play a role in this process. The functional targets have not yet been unequivocally identified by us, but we propose four potential candidates in this study. Although not all of these candidates showed interactions with BAY 60-6583 in molecular docking simulations, they are reported to be involved in T cell immune responses. Therefore, future experiments have to be conducted to find direct evidence that these proteins play a role in the CAR T cell-enhancing effects caused by BAY 60-6583.

In summary, we find that the small molecule BAY 60-6583 enhances CAR T cell activities relevant to the treatment of solid tumors, independently of the adenosine A2b receptor. We believe that BAY 60-6583 and its new potential targets that were identified in this study can be important clues for developing a novel immunotherapeutic strategy to enhance CAR T cell functions in cancer treatment.

## Data Availability

The raw data supporting the conclusions of this article will be made available by the authors, without undue reservation.
