# Supplementary material for: BAY 60-6583 Enhances the Antitumor Function of Chimeric Antigen Receptor-Modified T Cells Independent of the Adenosine A2b Receptor
Source: Front Pharmacol. 2021 Mar 12;12:619800. doi: 10.3389/fphar.2021.619800 (PMC7994267; doi:10.3389/fphar.2021.619800)
Supplement: Supplementary file 1 [file datasheet1.docx]

Supplementary Material


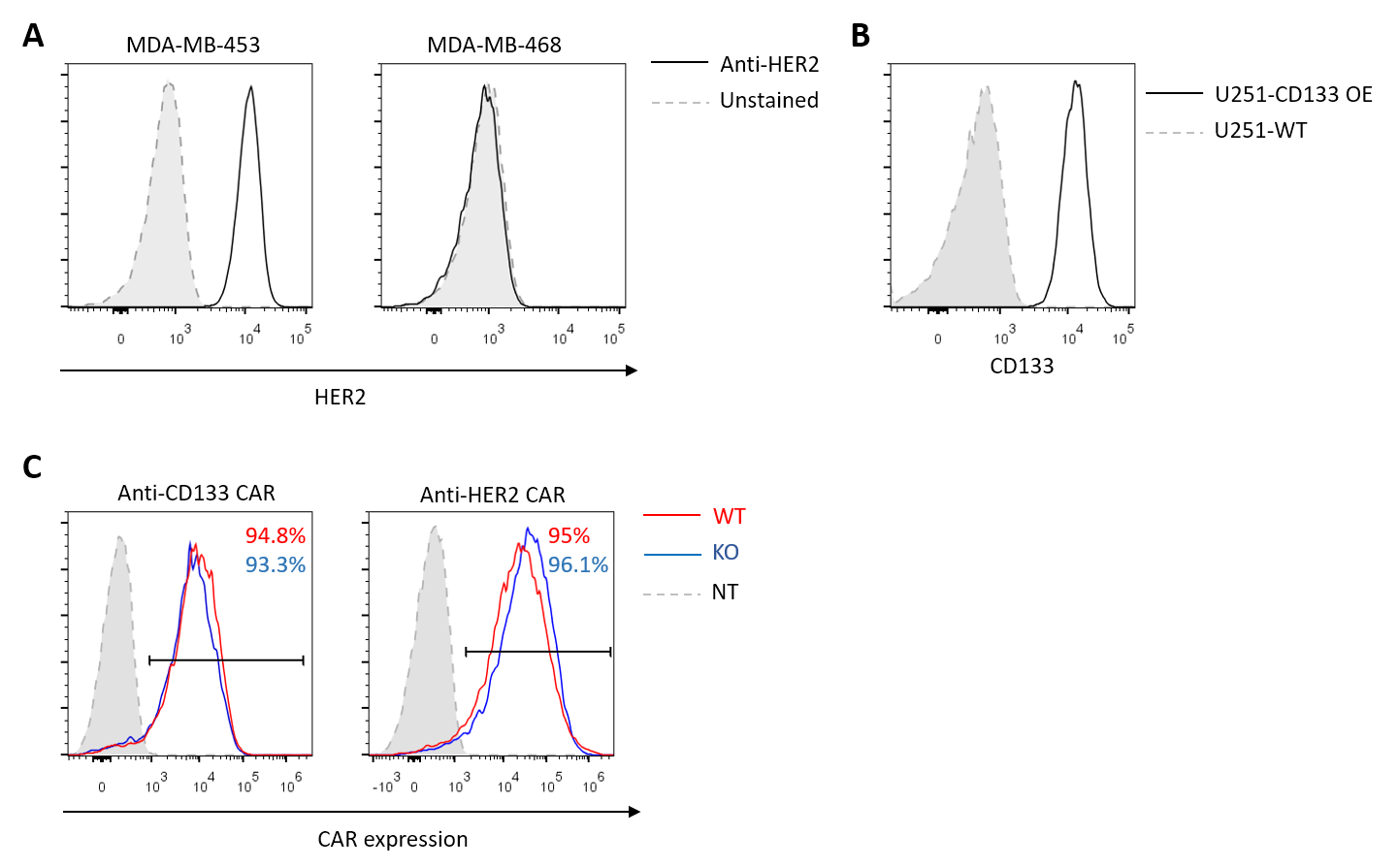


**Supplementary Figure 1. CAR expression on CAR T cells and antigen expression on target tumor cells.**

**(A)** Flow cytometry analysis of HER2 expression on the MDA-MB-453 and MDA-MB-468 cells. **(B)** Flow cytometry analysis of CD133 expression on the U251-CD133OE and U251-WT cells. **(C)** CAR expression on wild-type (WT) and adenosine A2b receptor knockout (KO) CAR T cells was determined according to flow-cytometric detection. Cells were stained by PE-conjugated anti-c-Myc antibody to determine expression of the CAR; non-transfected (NT) cells were used as negative control. Data shown are representative of several independent experiments.

**
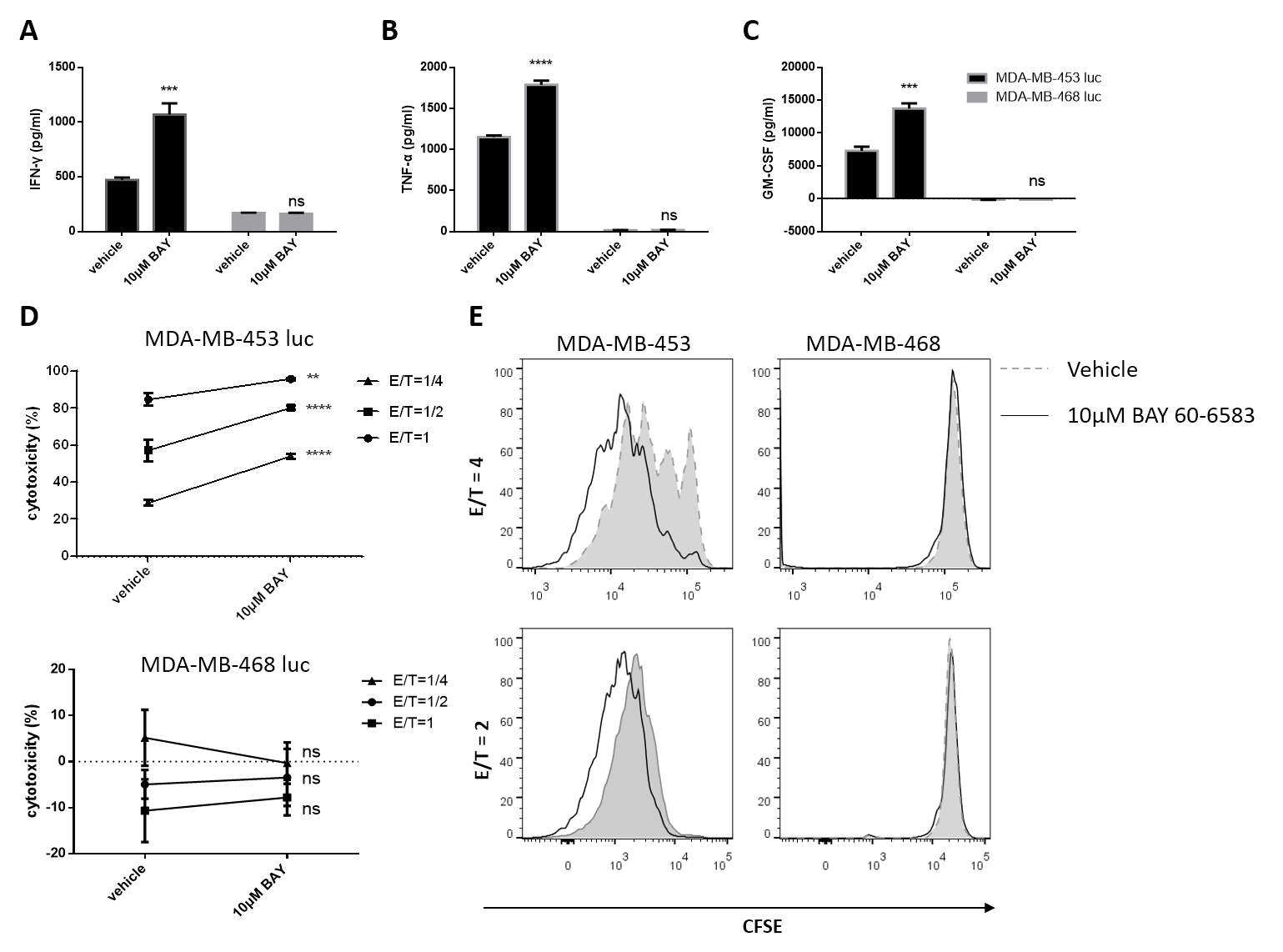
Supplementary Figure 2. BAY 60-6583 specifically improves activities of anti-HER2 CAR T cells.**

**(A-D)** Anti-HER2 CAR T cells were co-cultured with HER2-positive MDA-MB-453 luc cells or HER2-negative MDA-MB-468 luc cells at different E:T ratios. Co-cultures were performed in the presence of vehicle and BAY 60-6583. After 16 h, the medium was collected and the secretion of INF-γ **(A)**, TNF-α **(B)**, and GM-CSF **(C)** was detected using AlphaLISA kits (results for an E:T ratio of 1:2 are shown). **(D)** After 24 h of co-culture, cytotoxicity was determined by detecting the bioluminescence signal. Data are represented as the mean ± SD of triplicates from a representative experiment of *n* = 4 experiments. ****p*< 0.001, *****p*< 0.0001 by 2-way ANOVA; ns, not significant. **(E)** CFSE-labeled CAR T cells were co-cultured with irradiated HER2-positive MDA-MB-453 cells in the presence of BAY 60-6583 and vehicle control. After 120 h, flow cytometry was used to analyze cell proliferation. Results shown are from a representative experiment of *n* = 3 experiments.

**Supplementary**
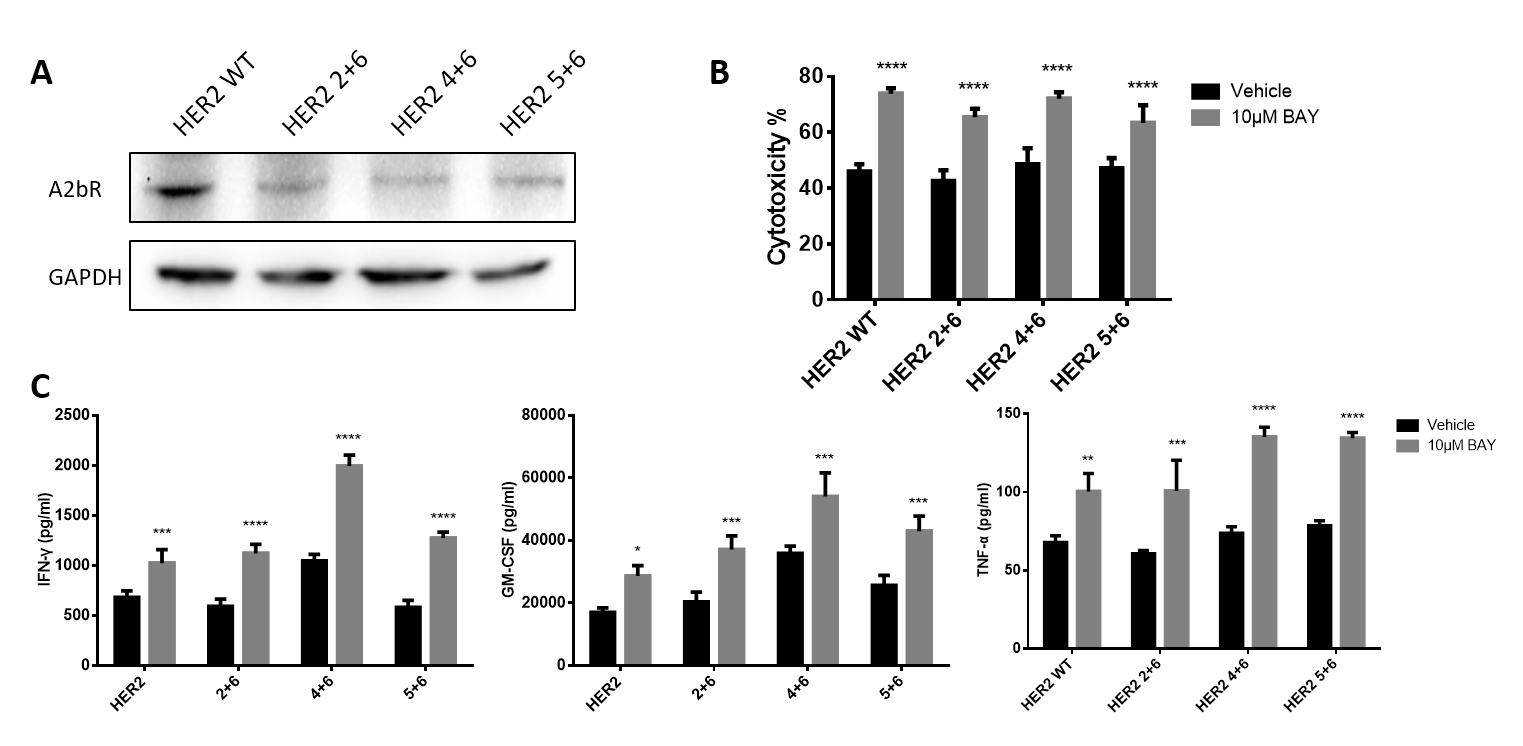
**Figure 3. BAY 60-6583-mediated enhanced antitumor activities were not suppressed in adenosine A2b receptor-deficient anti-HER2 CAR T cells.**

**(A)** The expression of the adenosine A2b receptor on WT and A2b receptor knockout anti-HER2 CAR T cells (2+6, 4+6, 5+6) was determined by Western blot. Data from a representative experiment of *n* = 3 experiments are shown. **(B and C)** WT and A2b receptor knockout anti-HER2 CAR T cells were co-cultured with HER2-positive MDA-MB-453 luc cells at an E:T ratio of 1:2. After treatment with vehicle control or BAY 60-6583 for 24 h, cytokine secretion in the supernatant was detected **(C)**; 48 h later, cytotoxicity was assessed **(B)**. **p*< 0.05, ****p*< 0.001 *****p*< 0.0001 by 2-way ANOVA. Data are represented as the mean ± SD from a representative experiment of *n* = 3 experiments.

| UniprotKB  ID | Protein name | Protein structure source | RCSB ID | Predicted binding score | Function |
| --- | --- | --- | --- | --- | --- |
| P35269 | General transcription factor IIF subunit 1 | N.A.^a^ | N.A. | N.A. | Required at transcription reaction (Rossignol et al., 1999). |
| Q9Y490 | Talin-1 | Experiment^b^ | 6R9T | –5.805 | Involved in the early assembly of adhesion (Manevich et al., 2007; Bluggel et al., 2011). Influences T helper cell differentiation (Meli et al., 2016). |
| P62263 | 40S ribosomal protein S14 | Modeled^c^ | N.A. | –4.196 | Required for 40S ribosomal subunit maturation (Wu et al., 2010). |
| P13796 | Plastin-2 | Modeled | N.A. | –4.695 | Supports T cell activation (Wabnitz et al., 2007), synaptic maturation (Morley, 2013). |
| P04350 | Tubulin beta-4A chain | Modeled | N.A. | –6.202 | The subunits of microtubules control the properties and functions of the microtubule (Brouhard and Rice, 2018; Janke and Magiera, 2020). |
| Q13885 | Tubulin beta-2A chain | Modeled | N.A. | –6.426 |  |
| Q9WUA3 | ATP-dependent 6-phosphofructokinase, platelet type | Experiment | 4XYJ | –4.598 | Extensive phosphate receptors: adenosine can be accepted (Hansen and Schonheit, 2001). |
| Q1XH10 | SKI/DACH domain-containing protein 1 | N.A. | N.A. | N.A. | Unknown |
| Q2NKX8 | DNA excision repair protein ERCC-6-like | N.A. | N.A. | N.A. | Important for intense cell proliferation (Albers et al., 2018). |
| Q9UMQ6 | Calpain-11 | Modeled | N.A. | –6.248 | May play an important role in the development and function of spermatogenic cells (Dear et al., 1999). |
| P16401 | Histone H1.5 | Modeled | N.A. | –4.86 | Marks splice sites (Glaich et al., 2019).Regulates gene expression during differentiation (Li et al., 2012). |
| P35579 | Myosin-9 | N.A. | N.A. | N.A. | Involved in cytokinesis, cellular polarity (Betapudi, 2010). |
| P42167 | Lamina-associated polypeptide 2 | N.A. | N.A. | N.A. | Participates in nuclear assembly (Martins et al., 2003) and chromatin structure stabilization (Dechat et al., 2000).  Cleaved into thymopoietin with immunomodulatory properties (Goldstein and Audhya, 1985; Gonser et al., 1999). |
| P62736 | Actin, aortic smooth muscle | Modeled | N.A. | –5.074 | Maintains contraction function of smooth muscle cells (Milewicz et al., 2017). |
| P14618 | Pyruvate kinase M (PKM) | Experiment | RCSB:4QG8 | –5.543 | Induces caspase-independent cell death (Stetak et al., 2007). Regulates the differentiation and development of Th1 and Th17 cells (Kono et al., 2019; Angiari et al., 2020). Induces expression of the immune checkpoint protein PD-L1 (Palsson-McDermott et al., 2017; Deng et al., 2018). |
| P08758 | Annexin A5 | Experiment | RCSB:1HAK | –-5.765 | Blood coagulation, apoptosis, phagocytosis (van Genderen et al., 2008). Membrane repair and virus docking (Lizarbe et al., 2013). |
| P60709 | Actin, cytoplasmic 1 | Experiment | RCSB:6ANU | –4.51 | Cytoskeletal structural proteins play a key role in most cellular processes (Guo et al., 2013). |
| Q9HB71 | Calcyclin-binding protein | Modeled | N.A. | –4.432 | Participates in cell proliferation, differentiation (Lian et al., 2019), protein dephosphorylation and ubiquitination (Goral et al., 2018). |

**Supplementary Table 1.** **The computational results for BAY 60-6583 and 18 candidate target proteins.**

^a^ Not available.

^b^ Its structure has been experimentally determined.

^c^ The structure was modeled using ITASSER.

**References：**

Albers, E., Sbroggio, M., Pladevall-Morera, D., Bizard, A.H., Avram, A., Gonzalez, P., et al. (2018). Loss of PICH Results in Chromosomal Instability, p53 Activation, and Embryonic Lethality. *Cell Rep* 24(12)**,** 3274-3284. doi: 10.1016/j.celrep.2018.08.071.

Angiari, S., Runtsch, M.C., Sutton, C.E., Palsson-McDermott, E.M., Kelly, B., Rana, N., et al. (2020). Pharmacological Activation of Pyruvate Kinase M2 Inhibits CD4(+) T Cell Pathogenicity and Suppresses Autoimmunity. *Cell Metab* 31(2)**,** 391-405.e398. doi: 10.1016/j.cmet.2019.10.015.

Betapudi, V. (2010). Myosin II motor proteins with different functions determine the fate of lamellipodia extension during cell spreading. *PLoS One* 5(1)**,** e8560. doi: 10.1371/journal.pone.0008560.

Bluggel, M., Spertini, F., Lutter, P., Wassenberg, J., Audran, R., Corthesy, B., et al. (2011). Toward protein biomarkers for allergy: CD4+ T cell proteomics in allergic and nonallergic subjects sampled in and out of pollen season. *J Proteome Res* 10(4)**,** 1558-1570. doi: 10.1021/pr100939g.

Brouhard, G.J., and Rice, L.M. (2018). Microtubule dynamics: an interplay of biochemistry and mechanics. *Nat Rev Mol Cell Biol* 19(7)**,** 451-463. doi: 10.1038/s41580-018-0009-y.

Dear, T.N., Moller, A., and Boehm, T. (1999). CAPN11: A calpain with high mRNA levels in testis and located on chromosome 6. *Genomics* 59(2)**,** 243-247. doi: 10.1006/geno.1999.5859.

Dechat, T., Vlcek, S., and Foisner, R. (2000). Review: lamina-associated polypeptide 2 isoforms and related proteins in cell cycle-dependent nuclear structure dynamics. *J Struct Biol* 129(2-3)**,** 335-345. doi: 10.1006/jsbi.2000.4212.

Deng, W., Zhu, S., Zeng, L., Liu, J., Kang, R., Yang, M., et al. (2018). The Circadian Clock Controls Immune Checkpoint Pathway in Sepsis. *Cell Rep* 24(2)**,** 366-378. doi: 10.1016/j.celrep.2018.06.026.

Glaich, O., Leader, Y., Lev Maor, G., and Ast, G. (2019). Histone H1.5 binds over splice sites in chromatin and regulates alternative splicing. *Nucleic Acids Res* 47(12)**,** 6145-6159. doi: 10.1093/nar/gkz338.

Goldstein, G., and Audhya, T.K. (1985). Thymopoietin to thymopentin: experimental studies. *Surv Immunol Res* 4 Suppl 1**,** 1-10. doi: 10.1007/bf02919050.

Gonser, S., Weber, E., and Folkers, G. (1999). Peptides and polypeptides as modulators of the immune response: thymopentin--an example with unknown mode of action. *Pharm Acta Helv* 73(6)**,** 265-273. doi: 10.1016/s0031-6865(99)00005-9.

Goral, A., Bartkowska, K., Djavadian, R.L., and Filipek, A. (2018). CacyBP/SIP, a Hsp90 binding chaperone, in cellular stress response. *Int J Biochem Cell Biol* 99**,** 178-185. doi: 10.1016/j.biocel.2018.04.012.

Guo, C., Liu, S., Wang, J., Sun, M.Z., and Greenaway, F.T. (2013). ACTB in cancer. *Clin Chim Acta* 417**,** 39-44. doi: 10.1016/j.cca.2012.12.012.

Hansen, T., and Schonheit, P. (2001). Sequence, expression, and characterization of the first archaeal ATP-dependent 6-phosphofructokinase, a non-allosteric enzyme related to the phosphofructokinase-B sugar kinase family, from the hyperthermophilic crenarchaeote Aeropyrum pernix. *Arch Microbiol* 177(1)**,** 62-69. doi: 10.1007/s00203-001-0359-1.

Janke, C., and Magiera, M.M. (2020). The tubulin code and its role in controlling microtubule properties and functions. *Nat Rev Mol Cell Biol*. doi: 10.1038/s41580-020-0214-3.

Kono, M., Maeda, K., Stocton-Gavanescu, I., Pan, W., Umeda, M., Katsuyama, E., et al. (2019). Pyruvate kinase M2 is requisite for Th1 and Th17 differentiation. *JCI Insight* 4(12). doi: 10.1172/jci.insight.127395.

Li, J.Y., Patterson, M., Mikkola, H.K., Lowry, W.E., and Kurdistani, S.K. (2012). Dynamic distribution of linker histone H1.5 in cellular differentiation. *PLoS Genet* 8(8)**,** e1002879. doi: 10.1371/journal.pgen.1002879.

Lian, Y.F., Huang, Y.L., Zhang, Y.J., Chen, D.M., Wang, J.L., Wei, H., et al. (2019). CACYBP Enhances Cytoplasmic Retention of P27(Kip1) to Promote Hepatocellular Carcinoma Progression in the Absence of RNF41 Mediated Degradation. *Theranostics* 9(26)**,** 8392-8408. doi: 10.7150/thno.36838.

Lizarbe, M.A., Barrasa, J.I., Olmo, N., Gavilanes, F., and Turnay, J. (2013). Annexin-phospholipid interactions. Functional implications. *Int J Mol Sci* 14(2)**,** 2652-2683. doi: 10.3390/ijms14022652.

Manevich, E., Grabovsky, V., Feigelson, S.W., and Alon, R. (2007). Talin 1 and paxillin facilitate distinct steps in rapid VLA-4-mediated adhesion strengthening to vascular cell adhesion molecule 1. *J Biol Chem* 282(35)**,** 25338-25348. doi: 10.1074/jbc.M700089200.

Martins, S., Eikvar, S., Furukawa, K., and Collas, P. (2003). HA95 and LAP2 beta mediate a novel chromatin-nuclear envelope interaction implicated in initiation of DNA replication. *J Cell Biol* 160(2)**,** 177-188. doi: 10.1083/jcb.200210026.

Meli, A.P., Fontes, G., Avery, D.T., Leddon, S.A., Tam, M., Elliot, M., et al. (2016). The Integrin LFA-1 Controls T Follicular Helper Cell Generation and Maintenance. *Immunity* 45(4)**,** 831-846. doi: 10.1016/j.immuni.2016.09.018.

Milewicz, D.M., Prakash, S.K., and Ramirez, F. (2017). Therapeutics Targeting Drivers of Thoracic Aortic Aneurysms and Acute Aortic Dissections: Insights from Predisposing Genes and Mouse Models. *Annu Rev Med* 68**,** 51-67. doi: 10.1146/annurev-med-100415-022956.

Morley, S.C. (2013). The actin-bundling protein L-plastin supports T-cell motility and activation. *Immunol Rev* 256(1)**,** 48-62. doi: 10.1111/imr.12102.

Palsson-McDermott, E.M., Dyck, L., Zaslona, Z., Menon, D., McGettrick, A.F., Mills, K.H.G., et al. (2017). Pyruvate Kinase M2 Is Required for the Expression of the Immune Checkpoint PD-L1 in Immune Cells and Tumors. *Front Immunol* 8**,** 1300. doi: 10.3389/fimmu.2017.01300.

Rossignol, M., Keriel, A., Staub, A., and Egly, J.M. (1999). Kinase activity and phosphorylation of the largest subunit of TFIIF transcription factor. *J Biol Chem* 274(32)**,** 22387-22392. doi: 10.1074/jbc.274.32.22387.

Stetak, A., Veress, R., Ovadi, J., Csermely, P., Keri, G., and Ullrich, A. (2007). Nuclear translocation of the tumor marker pyruvate kinase M2 induces programmed cell death. *Cancer Res* 67(4)**,** 1602-1608. doi: 10.1158/0008-5472.Can-06-2870.

van Genderen, H.O., Kenis, H., Hofstra, L., Narula, J., and Reutelingsperger, C.P. (2008). Extracellular annexin A5: functions of phosphatidylserine-binding and two-dimensional crystallization. *Biochim Biophys Acta* 1783(6)**,** 953-963. doi: 10.1016/j.bbamcr.2008.01.030.

Wabnitz, G.H., Kocher, T., Lohneis, P., Stober, C., Konstandin, M.H., Funk, B., et al. (2007). Costimulation induced phosphorylation of L-plastin facilitates surface transport of the T cell activation molecules CD69 and CD25. *Eur J Immunol* 37(3)**,** 649-662. doi: 10.1002/eji.200636320.

Wu, G.F., Hou, Y.L., Hou, W.R., Song, Y., and Zhang, T. (2010). Giant panda ribosomal protein S14: cDNA, genomic sequence cloning, sequence analysis, and overexpression. *Genet Mol Res* 9(4)**,** 2004-2015. doi: 10.4238/vol9-4gmr899.
